# Body Weight Distribution and Balance in Patients with Valgus or Varus Knee Deformity Treated with Hemiepiphysiodesis

**DOI:** 10.3390/jcm14238601

**Published:** 2025-12-04

**Authors:** Paweł Leyko, Monika Zaborska, Agnieszka Walczak, Łukasz Tomczyk, Marcin Pelc, Aleksander Mnich, Igor Kowal, Piotr Morasiewicz

**Affiliations:** 1 Department of Orthopaedic and Trauma Surgery, Institute of Medical Sciences, University of Opole, Al. Witosa 26, 45-401 Opole, Poland; 2 Faculty of Medicine, Institute of Medical Sciences, University of Opole, 45-040 Opole, Poland; 3 University Clinical Centre in Gdańsk, 80-952 Gdańsk, Poland; 4 Department of Food Safety and Quality Management, Poznan University of Life Sciences, Wojska Polskiego 31, 60-624 Poznań, Poland; 5 Institute of Medical Sciences, University of Opole, Oleska 48, 45-052 Opole, Poland; 6 Department of Orthopaedic and Trauma Surgery, Multidisciplinary Hospital in Zgorzelec, 59-900 Zgorzelec, Poland

**Keywords:** biomechanics, body load distribution, balance, hemiepiphysiodesis, knee deformity

## Abstract

**Background:** Biomechanical disorders may result from joint deformities. The purpose of this prospective research was to assess total load distribution over the lower limbs and balance in individuals before and after an hemiepiphysiodesis procedure performed due to valgus or varus knee deformity. **Methods:** Thirty-five patients, mean age 12 years, who underwent hemiphysiodesis for valgus or varus deformity of the knee were evaluated in comparison to a healthy control group. In patients, the percentage distribution of weight-bearing capacity between the operated and unoperated limbs was analyzed before and after surgery. Balance was assessed based on CoG (center of gravity) sway area and the CoG path length. Results were collected using the FreeMED MAXI pedobarographic platform. **Results:** Before surgery, statistically significant lower load on the entire affected limb was noted compared to unaffected limb. The values of path of center of gravity improved statistically significantly after surgery, compared to the values before surgery. There were no differences in the load on the treated lower limb in the study group and the non-dominant limb in the control group. There were no differences between the load on the non-operated limb in the study group and the load on the dominant limb in the control group. In the hemiepiphysiodesis group there were no significant differences between the mean total load on the treated and untreated limb after surgery. The median CoG sway area and path length in the group of patients after hemiphysiodesis and in the healthy control group did not differ. **Conclusions:** After hemiphysiodesis, the percentage load distribution did not differ between the operated and non-operated lower limb. Hemiepiphysiodesis allows for achieving balance similar to the healthy control group. Performing hemiepiphysiodesis allows for the improvement of balance parameters and load distribution in the lower limbs.

## 1. Introduction

Valgus or varus deformities, particularly of the knee joint, are relatively common in children [1,2,3,4,5,6]. Pain, joint instability, limited range of motion, limping, gait abnormalities, rapid development of joint degeneration, problems in playing sports, meniscal injuries, or aesthetic defects are issues that may be associated with knee deformities [7,8,9,10,11,12,13,14]. Moderate-to-severe valgus and varus knee deformities may adversely affect static and dynamic posture measurements [14,15,16,17,18,19,20]. Treatment indications for valgus or varus knee deformities are a mechanical axis deviation (MAD) of over 10 mm or an abnormal mechanical lateral distal femoral angle (mLDFA), or a mechanical medial proximal tibial angle (mMPTA) of over 10 degrees [1,3,5,6,7,11,12,14,16,20]. Unfortunately, in the case of knee deformities, conservative treatment, such as the use of medication, splints, exercises, or shoe inserts, is ineffective [1,2,3,4,5,6,7,8,20], with surgery being the only effective form of treatment for valgus or varus knee deformities [1,2,3,4,5,6,7]. Surgical techniques used for valgus or varus knee deformity correction include hemiepiphysiodesis (with O-plates, Blount staples, or screws) and osteotomy with either plate or external fixation [1,2,3,4,5,6,7,8,9,10,11,12,13,14,15,16,17,18,19,20,21,22].

Moderate-to-severe joint deformities, including those of the knee, may cause pain, diminish muscle strength, cause joint instability, and limit the range of motion, which may adversely affect the static lower-limb parameters of balance and body weight distribution [5,7,18,20,23,24]. Apart from deformity correction and pain relief, the purpose of hemiepiphysiodesis is to improve biomechanical parameters of the lower limbs [5,7,18,20]. Many authors suggest assessing various parameters, including biomechanical ones (including static ones—balance and percentage distribution of loads on the lower limbs) in patients with various pathologies of the musculoskeletal system [5,7,18,20,23,24,25,26,27,28,29]. Analysis of lower-limb biomechanical parameters, including balance and body weight distribution, helps objectively assess treatment outcomes and identify patients who may require longer and more intense rehabilitation [5,7,18,20,23,24,25,26,27,28,29].

The influence of hemiepiphysiodesis on the static parameters of the lower limbs in patients with knee deformities has not been adequately assessed or understood. There have been no studies of the effects of hemiepiphysiodesis in children with knee deformities on balance and body weight distribution over the lower limbs. There has been one study assessing gait parameters in patients after hemiepiphysiodesis due to knee deformity [20]. The authors observed no effect of knee deformity correction via hemiepiphysiodesis on most of the evaluated gait parameters [20]. Another study demonstrated improved balance and total body weight distribution after lower-limb corrective osteotomy with the Ilizarov method than before the procedure [23].

We posited a hypothesis that the use of hemiepiphysiodesis in the treatment of knee deformities in children would restore normal static parameters of the lower limbs. In this research, we aimed to prospectively assess the percentage distribution of lower limb loads and balance in patients before and after knee hemiphysiodesis and to compare the parameters of musculoskeletal statics with the healthy control group.

## 2. Materials and Methods

Prospective analysis of static biomechanical parameters of the lower limbs in patients with varus or valgus knee deformity who had undergone hemiepiphysiodesis at our department in the years 2021–2023. We established the following study inclusion criteria—distal femoral or proximal tibial hemiepiphysiodesis with a Pedi Plates O-Plate (Ortho Pediatrics, Warsaw, IN, USA) due to knee deformity (valgus or varus), complete medical and radiological records, complete data from balance and weight distribution assessments, age between 11 and 14 years, a lack of other lower limb pathology, no evidence of deformity recurrence at a clinical and radiological follow-up (in children before growth completion), a follow-up period of at least 1 year after treatment completion, and the patient’s assent. In all patients studied, the knee deformity (valgus or varus) was unilateral. The study exclusion criteria were patients aged under 11 years or over 14 years, a lack of patient assent, lower-limb comorbidities, incomplete clinical treatment records, incomplete data from balance and body weight distribution assessments, no history of hemiepiphysiodesis, bilateral knee deformity, and a follow-up period of less than 1 year after treatment completion. Our study had been approved by a local ethics committee (the first consent was concerned with data/research before the surgery; the second consent was concerned with data/research after the surgery) and was conducted in accordance with the Helsinki Declaration. The patients and their legal guardians had been informed of the voluntary nature of this study and the option of withdrawing from the study at any time. Taking into account the inclusion and exclusion criteria of the study, 35 eligible study subjects (21 males, 14 females) were selected for further analysis. The age of the patients was 11–14 years (mean age 12 years 5 months). The sex- and age-matched control group comprised 28 healthy volunteers, with a negative past medical history and no musculoskeletal abnormalities. The healthy control group comprised 17 males and 11 females, aged 11–14 years (a mean age of 12 years 6 months).

Surgical treatment was indicated based on clinical and radiological findings. The patients underwent proximal tibial or distal femoral hemiepiphysiodesis due to valgus or varus knee deformity. All patients evaluated underwent surgery using the same type of implants.

Radiological data were obtained from telemetric X-rays of the lower limbs. Telemetric X-rays were taken preoperatively and postoperatively at three-month intervals until deformity correction was achieved. After deformity correction, in patients who are still growing, a slight overcorrection of the deformity was attempted. In the period following deformity correction, radiographic follow-up was performed more frequently (every 1–2 months). In patients before surgery and during periodic postoperative control visits, MAD, mechanical medial proximal tibial angle, and mechanical lateral distal femoral angle were assessed based on telemetric X-rays of the lower limbs [1,12,15,16,19,20,21]. One of the evaluated parameters was mechanical axis deviation, defined as the distance between the mechanical axis of the lower limb and the center of the knee joint and expressed in millimeters [1,12,15,16,19,20].

Hemiepiphysiodesis was indicated based on the following criteria: a MAD of >10 mm and/or an inter-malleolar distance of >8 cm and/or a valgus or varus deformity of >10°, an age of 1–2 years prior to predicted growth completion, and no change in the extent of deformity on radiological follow-up at 3 months [1,3,5,6,7,11,12,14,16,19,20]. The implant was placed in the proximal tibia or the distal femur, depending on the location of the primary deformity, which was ascertained based on mLDFA and mMPTA anomalies [6,12,19,20]. All patients underwent hemiepiphysiodesis using the same technique by one of the same two surgeons. All operated patients had general anesthesia, and hemiepiphysiodesis was performed in the supine position. The site of O-Plate placement and the location of the growth plate were assessed via fluoroscopy. Subsequently, a 1–3 cm incision was made, and a PediPlates O-Plate was implanted under fluoroscopy to straddle the epiphyseal plate. On postoperative day one, the patients started walking with two elbow crutches. Weight-bearing capacity was gradually increased, based on pain tolerance, until full weight-bearing on the operated limb. Patients were admitted to the ward the day before surgery and discharged home the day after surgery. The first follow-up visits to the clinic took place 14 days after surgery for wound assessment and suture removal. Two weeks after surgery, patients were advised to discontinue crutches and walk with full weight-bearing on the operated limb. Two weeks after surgery, after suture removal, patients were allowed to return to school and to physical education classes and sports. Regular outpatient follow-up visits at the orthopedic clinic, including telemetric X-ray of the lower limbs and a clinical examination, took place every three months after surgery.

The O-Plate was removed either after achieving total deformity correction or after completing epiphyseal plate fusion. In patients still growing (with intact growth plates) in whom deformity correction was achieved, the O-Plate was removed only after growth completion (growth plate fusion) or after achieving a slight overcorrection of the deformity—in order to prevent deformity recurrence [2,7,19,20]. As recommended by other researchers, in patients with intact growth plates, the aim was to achieve a slight overcorrection of the deformity (MAD of up to 2 mm or 3–5 degrees) [2,7,19,20], with radiological follow-up every 1–2 months.

**Figure 1:**
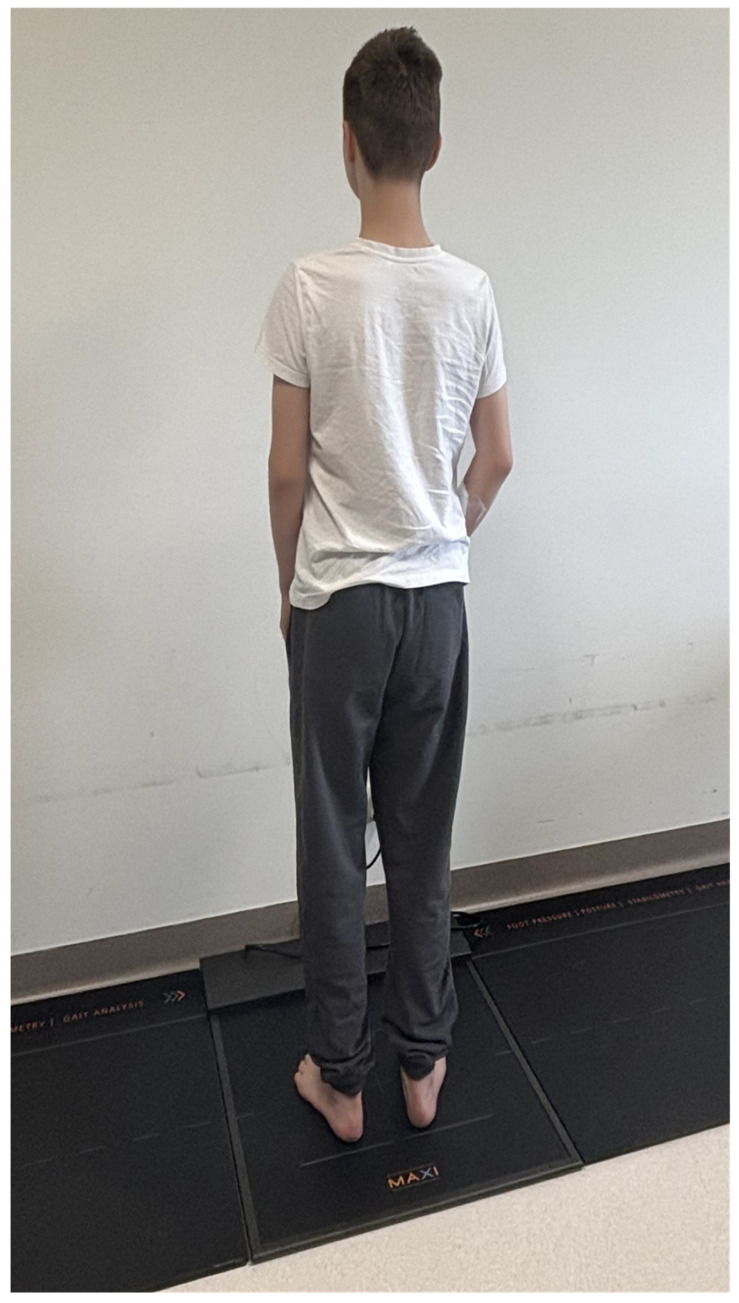
A patient on a pedobarographic platform.

Balance and total body weight distribution over the lower limbs were assessed with the use of a FreeMED MAXI pedobarographic platform, manufactured by SensorMedica (Guidonia Montecelio, Rome, Italy), Figure 1.

The pedobarographic assessment set included a 63.5 × 70 cm platform (active sensor total area of 50 × 60 cm), two inactive pads measuring 70 × 100 cm each, and a computer with appropriate software. The platform can sustain pressures of up to 150 N/cm^2^ in real time, with a minimum sampling frequency of 300 Hz. The 3000 square, 24-carat-gold-plated, resistive sensors, each with a durability of 1,000,000 cycles, ensure high measurement accuracy and reproducibility [24,25,26,27,28]. Pedobarographic platforms are used in assessing static biomechanical parameters of the lower limbs [23,24,25,26,27,28]. In patients before surgery and after hemiepiphysodesis, during long-term follow-up, the intensity of pain was assessed using a visual analog scale (VAS), where 0 meant no pain, and 10 meant maximum pain.

All study subjects received detailed information on the course of the assessment. During the assessment of balance and load distribution of the lower limbs using a tensometric mat (pedobarographic platform), all assessed patients from the study group and those from the control group were asked to maintain their posture. All subjects underwent assessments in their socks, in the same room, and on the same pedobarographic platform. Assessments were conducted with the patients standing on both feet, with their feet pointed comfortably forward (external rotation of 5–10°) and their eyes open. Weight distribution was recorded after a 5 s period of stabilization following the patient’s stepping onto the pedobarographic platform. All assessed patients from the study group and those from the control group were instructed to maintain an upright posture, with their arms hanging symmetrically along the torso, and keep their eyes fixed on at eye-level on the wall in front of them. All assessments of balance and load distribution of the lower limbs using a tensometric mat (pedobarographic platform) were conducted by the same researcher. The person performing pedobarographic measurements did not know which group the subjects belonged to.

Each subject underwent three assessments, and the mean value was used in further analyses. The collected data were saved with a specialist FreeSTEP software, version 2.02.006, designed to process data and calculate balance and load distribution parameters. The collected measurement data were exported to a spreadsheet and analyzed statistically.

**Figure 2:**
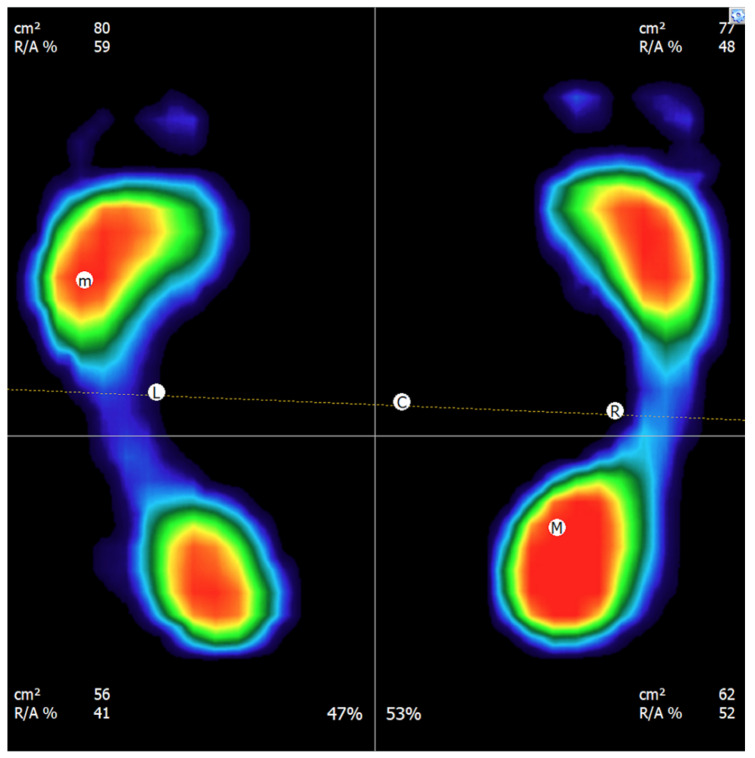
Example of the measurement result of the percentage distribution of loads on the lower limbs.

The following parameters were recorded and analyzed—forefoot load in the healthy lower limb [%], total load on the healthy lower limb [%], hindfoot load in the healthy lower limb [%], forefoot load in the treated lower limb [%], total load on the treated lower limb [%], and hindfoot load in the treated lower limb [%], Figure 2.

Balance was assessed based on the CoG sway area and the center of gravity (CoG) path length [23,25,26,28]. The CoG path length, which was expressed in millimeters, was defined as the total distance covered by the CoG during the assessment [23,25,26,28]. The CoG sway area, which was expressed in square millimeters, was determined by the maximum CoG sway and defined as the area limited by maximum CoG sway in all directions during the assessment [23,25,26,28].

These pedobarographic assessments were conducted before surgery, and in patients who had undergone surgical treatment, at least 12 months after implant removal. The healthy lower limb in the patient group was compared with the dominant lower limb in the control group, and the treated limb in the patient group was compared with the nondominant limb in the control group [28,29]. Total weight distribution values and balance parameters in patients were compared with those in healthy volunteers from the control group.

### Statistical Analysis

In the statistical analyses conducted for this study, procedures were selected to match both the characteristics of the data and the repeated-measures design. Prior to performing the main analyses, the normality of the variables and of the difference scores was assessed using the Shapiro–Wilk test, which is recommended for small to moderate sample sizes. This step allowed for verification of whether the distribution of the data met the assumptions required for the application of parametric methods.

After confirming that the normality assumption was satisfied, paired-samples *t*-tests were employed to compare the values obtained before and after the surgical intervention. This procedure enabled the evaluation of statistically significant differences in means between the two time points for the same participants. For each variable, the *t*-value, associated degrees of freedom, and two-tailed *p*-value were calculated. To assess the precision of the estimates, 95% confidence intervals were also determined for the mean differences. Additionally, irrespective of statistical significance, the effect size was quantified using Cohen’s d for paired samples, calculated based on the t-value and sample size (d = t/√n). Effect sizes were interpreted according to commonly accepted thresholds, where values around 0.20 indicate a small effect, around 0.50 a moderate effect, and values ≥ 0.80 a large effect. This allows assessment of the practical or clinical relevance of the findings beyond statistical significance.

To examine potential asymmetry in body-weight distribution between the operated and non-operated limb prior to surgery, independent-samples *t*-tests were performed. For these inter-limb comparisons, the t-value, degrees of freedom, and two-tailed *p*-value were calculated, and effect sizes were computed using Cohen’s d for independent groups. The same interpretative thresholds were applied to ensure consistency across analyses.

For stabilometric variables that were non-normally distributed and expressed using medians and quartiles, non-parametric comparisons between the patient and control groups were conducted using the Mann–Whitney U test. Alongside the Z statistic and *p*-value, the effect size r (r = |Z|/√N) was calculated. Based on standard guidelines, r values of approximately 0.10, 0.30, and 0.50 reflect small, medium, and large effects, respectively. For comparability with parametric analyses, an approximate conversion of r to Cohen’s d was also provided, recognizing that such transformations offer an estimate of effect size magnitude rather than a direct parametric equivalent.

All analyses were performed using the Statistica 14.1 software package, with the significance level set at α = 0.05. The results were interpreted in accordance with current standards for reporting statistical data in biomedical research.

No a priori sample size calculation was performed. The sample size was determined by the number of consecutive patients who met the inclusion criteria during the recruitment period; therefore, the statistical power of the study may be limited.

## 3. Results

The mean total load on the healthy lower limb in the patient after hemiphysiodesis and on the dominant lower limb in the control healthy group was 50.72% and 47.71%, respectively; the difference was not statistically significant (Table 1). The mean load on the treated lower limb in the patient after hemiepiphysiodesis and on the non-dominant lower limb in the control group was 49.57% and 52.28%, respectively; the difference was not statistically significant (Table 1). We observed no significant differences between the treated lower limb in the experimental group and the nondominant lower limb in the control healthy group in terms of forefoot or hindfoot loading (Table 1). Similarly, there were no differences between the healthy lower limb in the patient after hemiepiphysiodesis group and the dominant lower limb in the healthy control group in terms of either forefoot or hindfoot loading (Table 1).

In the hemiepiphysiodesis group, there were no significant differences between the mean total load on the treated and untreated lower limb (Table 2). The mean forefoot load in the treated and untreated lower limbs was 45% and 44%; the difference was not statistically significant (Table 2). In the same group, the mean hindfoot load in the treated and untreated lower limbs was 55% and 56%, respectively; the difference was not statistically significant (Table 2).

The median CoG path length was 1077.21 mm and 1146.31 mm in the experimental and control group, respectively; the difference was not statistically significant (Table 3).

The median CoG sway area was 132.58 mm^2^ and 118.66 mm^2^ in the experimental and control groups, respectively. These values did not differ significantly between the groups (Table 3). In the VAS pain intensity scale, none of the patients experienced pain before surgery (VAS = 0). During the long-term follow-up after hemiepiphysodesis, none of the patients experienced pain (VAS = 0).

**Figure 3:**
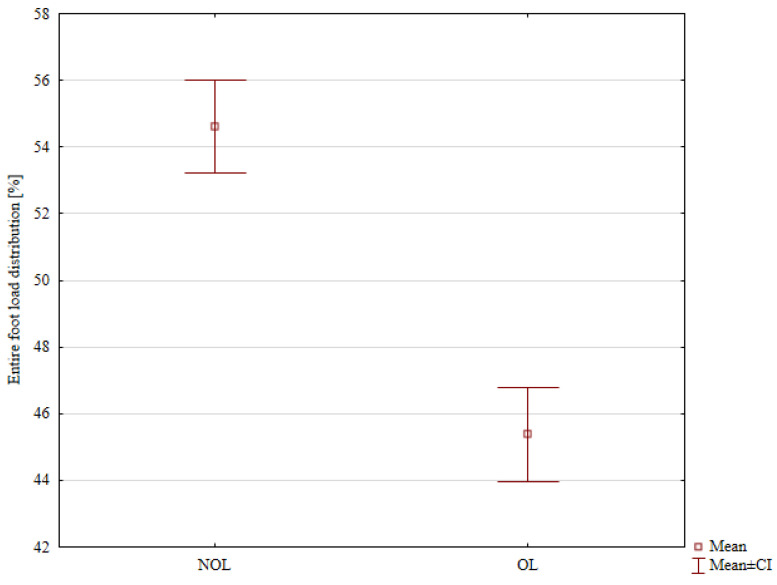
The entire foot load distribution in the Non-Operated Limb (NOL) and the Operated Limb (OL) before surgery.

Detailed results of the percentage distribution of loads on the patients’ lower limbs before hemiepiphysiodesis are presented in Table 4. There were no statistical differences in the distribution of loads on the forefoot and hindfoot between the affected and unaffected limbs. A statistically significant lower load on the entire affected limb was noted (mean 45.38%) compared to the load on the entire unaffected limb (mean 54.62%), *p* < 0.001, Figure 3.

**Figure 4:**
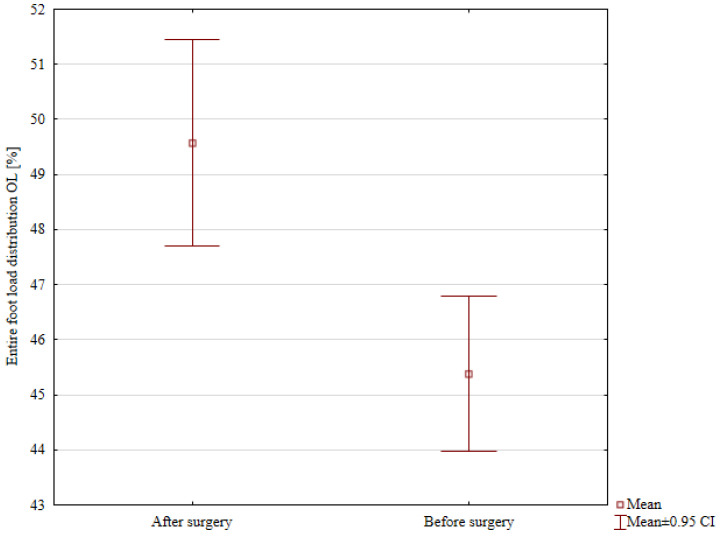
Comparison of the entire foot load distribution in the Operated Limb (OL) before and after surgery.

**Figure 5:**
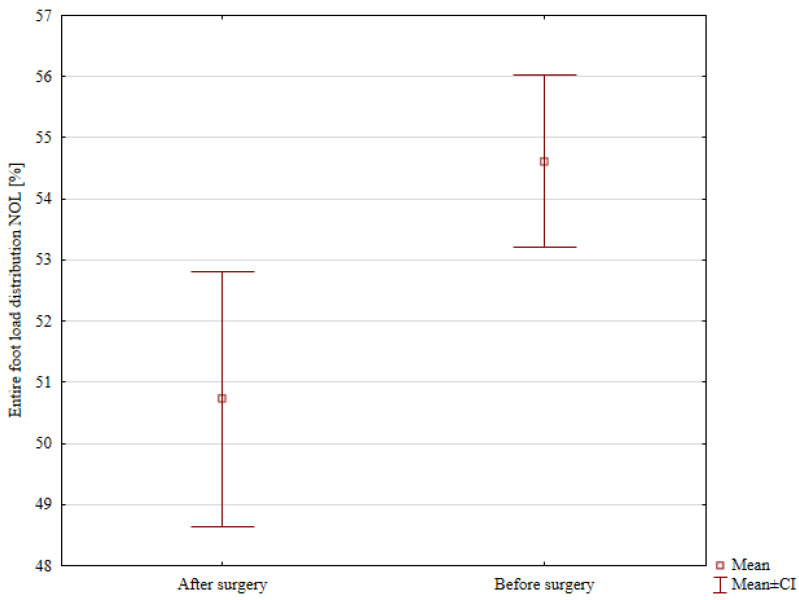
Comparison of the entire foot load distribution in the Non-Operated Limb (NOL) before and after surgery.

A detailed comparison of the percentage distribution of loads on the lower limbs of patients before and after hemiepiphysiodesis is presented in Table 5. The load on the entire operated lower limb before treatment was statistically significantly lower (mean 45.38%) compared to the value after treatment (mean 49.58%; *p* < 0.001; Figure 4). The total load on the unoperated lower limb statistically decreased from the mean value of 54.67% before surgery to the mean value of 50.73% after surgery (*p* = 0.007; Figure 5).

**Figure 6:**
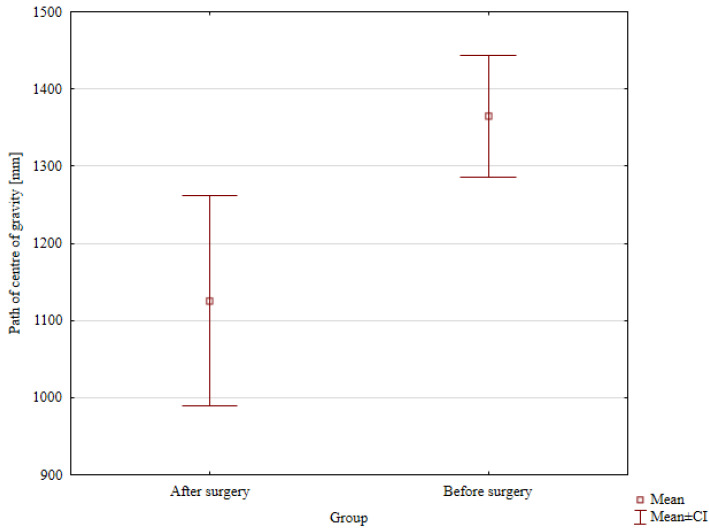
Comparison of the path of the center of gravity before and after surgery.

Detailed results of the comparison of patients’ balance parameters before and after hemiepiphysodesis are presented in Table 6. The values of the path of the center of gravity improved statistically significantly, from the mean value of 1360.84 mm before surgery to the mean value of 1125.51 mm after treatment (*p* = 0.049; Figure 6). The field area of the center of gravity values after treatment, compared to the values before treatment, improved, but without statistical significance.

## 4. Discussion

The purpose of this research was to assess balance and percentage distribution of body loads between lower limb parameters in patients who underwent hemiepiphysiodesis for varus or valgus knee deformity. Before the surgeries, we found a significantly lower percentage distribution of load on the affected limb compared to the unaffected one. The study showed no significant differences between the patient group after hemiepiphysiodesis and the healthy control group in terms of the assessed balance parameters. Total body weight distribution over the lower limbs showed no significant differences between the patient group after hemiepiphysiodesis and the healthy control group. Moreover, there were no differences between the treated and untreated limbs in the experimental group after surgery in terms of load distribution. The values of path of the center of gravity improved statistically significantly after surgery, compared to the values before surgery. The results of our study partially support our research hypothesis.

Lower limb deformities may lead to joint instability, muscle weakness, range of motion limitations, pain, meniscal damage, and ligament laxity [1,2,3,4,5,6,7,8,9,10,12,23,26,28], all of which can adversely affect body weight distribution and balance [5,6,7,18,20,23,26,28,29]. Normal lower-limb load distribution is essential for maintaining postural control [24]. The purpose of hemiepiphysiodesis is to partially or completely correct valgus or varus knee deformity [1,2,3,4,5,6,7,8,9,10,11,12,13,14,15,16,17,18,19,20,21,22], which should improve body weight distribution and balance.Previous studies on the use of hemiepiphysiodesis assessed mainly radiological and clinical parameters [1,2,3,4,5,6,7,8,9,10,12,13,14,16,17,18,19]. There was only one study that evaluated gait parameters following hemiepiphysiodesis [20]. In this study, researchers observed no differences between the patient group after hemiepiphysiodesis and the healthy control group in terms of gait cycle duration, stride length, stance phase duration, swing phase duration, double support duration, single support duration, gait speed, cadence, or step length [20]. Gait parameters after hemiphysiodesis did not improve compared to preoperative values [20].

There are no available reports on the analysis of the effect of hemiepiphysiodesis on balance and body weight distribution over the lower extremities. Biomechanical assessments, which include static parameters, are an important part of evaluating treatment outcomes in various musculoskeletal pathologies [5,7,18,20,23,26,28,29].

The use of hemiepiphysiodesis helps restore the normal mechanical axis of the lower limb, which should improve lower-limb biomechanical parameters, including static parameters [2,3,4,5,6,7,9,14,17,18]. According to Goldman and Green, valgus or varus knee deformity may produce ligament laxity, pain, joint instability, and a shift in joint load, which may adversely affect lower-limb biomechanics [5]. Other authors also suggest that due to ligamentous laxity and pain, knee deformities may impair lower-limb biomechanics [7,18]. On the other hand, some authors state that hemiepiphysiodesis procedures were performed in patients with mild-to-moderate deformities (severe deformities required osteotomy), which did not cause joint instability, pain, or ligament laxity [20]. Conversely, some researchers demonstrated no effect of hemiepiphysiodesis on gait parameters [20]. Those authors assessed 35 patients after a hemiepiphysiodesis procedure conducted to treat valgus or varus knee deformity and observed no changes in gait parameters following treatment in comparison with those measured at baseline [20]. The authors identified several factors that do not alter gait parameters following hemiepiphysiodesis [20]. The authors suggested that the age of the study population (a mean of 12 years) was responsible for the considerable compensatory capacity [20]. In the group of patients we assessed, we did not observe any pain before hemiepiphysiodesis, so it should be assumed that the knee joint deformities in the patients we assessed did not cause pain before surgery. During the postoperative follow-up assessment of the patients, we also did not observe any pain in any of the patients, which indicates a good treatment outcome. Courvoisier et al. report the absence of pain in all patients in the follow-up after hemiphysiodesis [3]. Brauwer and Moens reported pain in 16% of patients after hemiphysiodesis [8]. The improvement in balance parameters and the percentage distribution of lower-limb loads in our patients after surgery may have resulted from the correction of the mechanical axis of the lower limbs. The improvement in lower-limb static parameters after treatment may have been influenced by better joint stability, improved muscle strength, and proper joint mobility.

In one of the studies, the authors assessed the static parameters of the musculoskeletal system (percentage distribution of lower limb loads and balance) using a pedobarographic platform in 20 patients prior to and after corrective osteotomy with Ilizarov external fixation [23]. The authors observed symmetrical load distribution over the two lower limbs following treatment [23]. There was a post-treatment improvement in balance, with a CoG path length of 125.11 cm and a CoG sway area of 5.81 cm^2^ [23]. Arslan and Görgü assessed balance in 42 patients with juvenile idiopathic scoliosis [25]. Those authors reported improved balance in the group of patients with scoliosis who used insoles [25]. Brusa et al. used a pedobarographic platform to evaluate balance and body weight distribution in 19 patients with congenital hypothyroidism and a mean age of 10.52 years and compared the results with those from a healthy control group [26]. Those authors reported weight distribution asymmetry and worse balance in patients in comparison with the healthy control group [26]. Pedobarographic assessments were also used in another study to assess balance and body weight distribution in 57 patients who underwent corticotomy with the Ilizarov method and to compare the results with those of a healthy control group [28]. The authors observed no differences in weight distribution between the two groups; however, balance parameters were worse in the Ilizarov corticotomy group than in the control group [28]. In our study, balance measurement results were similar to those reported by other authors [23,24,25,28], which indicates measurement reproducibility.

In the available literature, we found no articles assessing static parameters (balance and percentage of lower limb load distribution) in patients before and after hemiepiphysiodesis. There were also no studies assessing lower-limb static parameters after hemiepiphysiodesis compared to a healthy control group. In our study, we observed improved balance parameters after hemiepiphysiodesis compared to preoperative results, indicating a positive impact on lower limb biomechanics and good treatment outcomes. Before hemiepiphysiodesis, the percentage distribution of lower limb loads was asymmetric (lower in the affected lower limb). In the follow-up after hemiepiphysiodesis, the percentage distribution of lower-limb loads did not differ between the operated and unoperated limbs, which further supports a positive assessment of our patients’ treatment outcomes.

In our study, the assessed specific parameters of total, forefoot, or hindfoot load distribution in patients who underwent treatment showed no significant differences between the treated and healthy limb after hemiepiphysiodesis. These data demonstrate that hemiphysiodesis does not cause negative effects on lower-limb function, based on the percentage distribution of lower-limb loads. Hemiepiphysiodesis helps achieve symmetrical load distribution over the lower limbs, which is associated with normal musculoskeletal static parameters [23,25,27,28] and indicates good treatment outcomes. We observed no differences between the group of hemiepiphysiodesis patients after surgery and the healthy control group in terms of total, forefoot, or hindfoot load distribution. Our study demonstrated no differences in balance between patients who underwent hemiepiphysiodesis and healthy individuals from the control group.

It is worth noting that our study population after treatment and the healthy control group showed no significant differences in static posturography parameters, which indicates restored lower-limb function following valgus or varus knee deformity with hemiepiphysiodesis. Atac et al., who assessed body weight distribution over the lower limbs of 30 healthy athletes, reported no differences between the lower limbs in terms of load distribution [24]. The mean percentage values of total, forefoot, and hindfoot load distribution obtained by Atac et al. [24] were comparable with those observed in our study population after treatment.

Our study showed that deformity correction yielded static posturography parameters similar to those in the healthy control group. This may indicate normal muscle strength, reduced pain, and normal joint mobility after treatment. Our study results suggest a lack of adverse effects of hemiepiphysiodesis in valgus or varus knee deformity correction on lower-limb biomechanics. All results and conclusions from our study should be considered with caution due to the small study and control groups.

There have been some reports that the etiology of knee deformities may affect the outcomes of hemiepiphysiodesis [1,4,5,7,11]; therefore, we excluded post-traumatic, post-inflammatory, neurogenic, and rachitic deformities from our study; instead, we evaluated only patients with idiopathic knee deformities. As reported by other researchers, the type of deformity (varus or valgus knee deformity) does not influence the clinical, radiological, and functional results in patients after hemiepiphysodesis [20,21].

One limitation of our study is a moderate sample size, which is due to our wide and relatively restrictive inclusion and exclusion criteria in order to ensure as homogeneous a study group as possible. The small study group size resulted from the lack of a large number of patients after hemiepiphysiodesis, and the desire to present interesting research results relatively quickly. Other authors also compared study populations that were of similar size or smaller than ours [3,8,9,10,13,18,20,22,23,24,25]. The strengths of our study are the use of the same surgery technique and rehabilitation protocols, and having the pedobarographic assessments conducted by the same researcher. A strength of our study was that hemiepiphysiodesis was performed in all patients by only one of the same two orthopedic surgeons. Other strengths of our study include comparing the results of patients with those of healthy controls, assessing only patients with idiopathic deformities, and a narrow age range (11–14-year-olds). Our next research goals include biomechanical studies of patients before and after hemiepiphysiodesis, with a follow-up period of several years. In the longer term, we also plan to collect and evaluate static and dynamic parameters in a larger group of patients after hemiepiphysiodesis using a pedobarographic platform.

The practical significance of this study is to provide information on the impact of hemiepiphysiodesis on one of the key aspects of assessing the outcomes of lower-limb pathology treatment: biomechanical parameters. Our results demonstrate that patients who underwent hemiepiphysiodesis have practically normal biomechanical parameters. Our results demonstrate that patients are on the correct treatment and rehabilitation algorithm, requiring no changes. Hemiepiphysiodesis improves lower-limb static parameters, confirming the indication for this type of surgery.

## 5. Conclusions

After hemiphysiodesis, the percentage load distribution did not differ between the operated and non-operated lower limb.

We observed no differences between the patient and healthy control groups either in terms of balance or body weight distribution over the lower limbs.

Hemiepiphysiodesis allows for improvement of balance parameters and percentage distribution of lower-limb loads after treatment, compared to pre-operative values.

The treatment outcomes in terms of static posturography parameters following valgus or varus knee deformity correction with hemiepiphysiodesis were good.

Conclusions from our research should be considered with caution due to the small group size.

## Figures and Tables

**Table 1:** Body weight distribution of the patients compared to the control group.

Variable	Patients (Mean ± SD)	Control Group (Mean ± SD)	Mean Difference	t	df	*p*	95% CI	Cohen’s d
Entire foot load NOL [%]	50.72 ± 5.88	47.71 ± 6.89	3.01	1.53	45	0.134	−0.96–6.99	0.43
Forefoot load NOL [%]	44.00 ± 10.36	42.43 ± 11.53	1.57	0.46	45	0.648	−5.38–8.52	0.13
Hindfoot load NOL [%]	56.00 ± 10.36	56.14 ± 13.59	−0.14	−0.04	45	0.969	−7.60–7.32	0.01
Entire foot load OL [%]	49.58 ± 5.30	52.29 ± 6.89	−2.71	−1.46	45	0.150	−6.42–1.01	0.41
Forefoot load OL [%]	45.06 ± 9.48	45.57 ± 12.01	−0.51	−0.16	45	0.877	−7.03–6.02	0.04
Hindfoot load OL [%]	54.93 ± 9.48	53.29 ± 11.99	1.65	0.50	45	0.616	−4.99–8.29	0.14

Note. OL = Operated Limb; NOL = Non-Operated Limb; SD = Standard Deviation. *p* = *p*-value (Student’s *t*-test for independent samples); 95% CI = 95% confidence interval for the mean difference; Cohen’s d = effect size for paired samples, calculated as d = t/√n.

**Table 2:** Percentage distribution of body loads between lower limbs in the group of patients after hemiphysiodesis and in the healthy control group.

Control Group								
Variable	NOL (Mean ± SD)	OL (Mean ± SD)	Mean Diff (OL–NOL)	t	df	*p*	95% CI	Cohen’s d
Entire foot load [%]	47.71 ± 6.90	52.29 ± 6.90	4.57	−1.75	26	0.091	−9.95–0.81	0.64
Forefoot load [%]	42.43 ± 11.53	45.57 ± 12.01	3.14	−0.71	26	0.486	−6.00–12.29	0.25
Hindfoot load [%]	56.14 ± 13.60	53.29 ± 12.00	−2.86	0.59	26	0.561	−6.66–12.38	0.21
Patients after surgery								
Entire foot load [%]	50.72 ± 5.88	49.58 ± 5.30	−1.15	0.84	64	0.406	−1.56–3.86	0.15
Forefoot load [%]	44.00 ± 10.36	45.06 ± 9.48	1.06	−0.43	64	0.666	−3.98–6.11	0.08
Hindfoot load [%]	56.00 ± 10.36	54.94 ± 9.48	−1.06	0.43	64	0.666	−3.99–6.12	0.08

Note. OL = Operated Limb; NOL = Non-Operated Limb; SD = Standard Deviation. *p* = *p*-value (Student’s *t*-test for independent samples); 95% CI = 95% confidence interval for the mean difference; Cohen’s d = effect size for paired samples, calculated as d = t/√n.

**Table 3:** Path of the center of gravity and field area of the center of gravity in patients after hemiepiphysiodesis and in the healthy control group.

Analyzed Variable	Group	Q1	Median	Q3	Z	*p*	Effect Size r	Approx. Cohen’s d
Path of center of gravity [mm]	Patients	831.55	1077.21	1386.57	0.0368	0.971	0.0065	0.013
	Control group	788.49	1146.32	1281.35				
Field area of the center of gravity [mm^2^]	Patients	60.38	132.58	282.30	0.2329	0.816	0.041	0.082
	Control group	37.86	118.66	559.16				

Note. Z—standardized statistic for the Mann–Whitney U test; *p*—*p*-value for the Mann–Whitney U test; Q1, Q3—first and third quartiles; r—effect size for Mann–Whitney (r = |Z|/√N); d—approximate conversion from r → Cohen’s d = 2r/√(1 − r^2^)); interpret with caution (non-parametric).

**Table 4:** Percentage distribution of body loads between lower limbs in the group of patients before hemiphysiodesis.

Variable	NOL (Mean ± SD)	OL (Mean ± SD)	Mean Difference	t	df	*p*	95% CI	Cohen’s d
Entire foot [%]	54.62 ± 4.03	45.38 ± 4.03	9.45	9.45	66	<0.001	7.45–11.45	2.30
Forefoot [%]	42.85 ± 10.32	45.38 ± 9.19	−2.53	−1.07	66	0.290	−7.25–2.19	0.26
Hindfoot [%]	56.56 ± 11.26	54.15 ± 9.21	2.41	0.97	66	0.337	−2.53–7.35	0.24

Note. OL = Operated Limb; NOL = Non-Operated Limb. SD = Standard Deviation; *p* = *p*-value (Student’s *t*-test for independent samples); 95% CI = 95% confidence interval for the mean difference; Cohen’s d = effect size for paired samples, calculated as d = t/√n.

**Table 5:** Body weight distribution for the patients before and after surgery.

Variable	Before Surgery (Mean ± SD)	After Surgery (Mean ± SD)	Mean Difference	t	df	*p*	95% CI	Cohen’s d
Entire foot OL [%]	45.38 ± 4.03	49.58 ± 5.30	4.24	4.89	32	<0.001	3.15–7.64	0.85
Forefoot OL [%]	45.38 ± 9.19	45.06 ± 9.48	−0.32	−0.60	32	0.550	−5.56–3.02	0.10
Hindfoot OL [%]	54.15 ± 9.21	54.94 ± 9.48	0.70	0.32	32	0.748	−2.58–6.10	0.06
Entire foot NOL [%]	54.67 ± 4.08	50.73 ± 5.88	−3.94	−2.87	32	0.007	−6.74–1.14	0.5
Forefoot NOL [%]	42.85 ± 10.32	44.00 ± 10.36	1.12	0.44	32	0.662	−4.06–6.30	0.08
Hindfoot NOL [%]	56.56 ± 11.26	56.00 ± 10.36	−0.52	−0.19	32	0.849	−5.98–4.95	0.03

Note. OL = operated limb; NOL = non-operated limb; SD = standard deviation; *p* = *p*-value from paired-samples *t*-test; 95% CI = 95% confidence interval for the mean difference; Cohen’s d = effect size for paired samples, calculated as d = t/√n.

**Table 6:** Path of the center of gravity and field area of the center of gravity in patients before and after surgery.

Variable	Before Surgery (Mean ± SD)	After Surgery (Mean ± SD)	Mean Difference	t	df	*p*	95% CI	Cohen’s d
Path of center of gravity [mm]	1360.84 ± 225.36	1125.51 ± 371.60	−235.33	−3.04	30	0.0049	−393.56 to −77.09	−0.55
Field area of the center of gravity [mm^2^]	385.53 ± 294.37	255.56 ± 343.40	−129.97	−1.42	30	0.1653	−316.60 to 56.66	−0.26

Note. SD = standard deviation; *p* = *p*-value from paired-samples *t*-test; 95% CI = 95% confidence interval for the mean difference; Cohen’s d = effect size for paired samples, calculated as d = t/√n.

## Data Availability

The data presented in this study are available on request from the corresponding author.

## References

[1] Trisolino G., Boarini M., Mordenti M., Evangelista A., Gallone G., Stallone S., Zarantonello P., Antonioli D., Di Gennaro G.L., Stilli S. (2021). Outcomes of Temporary Hemiepiphyseal Stapling for Correcting Genu Valgum in Children with Multiple Osteochondromas: A Single Institution Study. Children.

[2] Castañeda P., Urquhart B., Sullivan E., Haynes R.J. (2008). Hemiepiphysiodesis for the correction of angular deformity about the knee. J. Pediatr. Orthop..

[3] Courvoisier A., Eid A., Merloz P. (2009). Epiphyseal stapling of the proximal tibia for idiopathic genu valgum. J. Child. Orthop..

[4] Dai Z.Z., Liang Z.P., Li H., Ding J., Wu Z.K., Zhang Z.M., Li H. (2021). Temporary hemiepiphysiodesis using an eight-plate implant for coronal angular deformity around the knee in children aged less than 10 years: Efficacy, complications, occurrence of rebound and risk factors. BMC Musculoskelet. Disord..

[5] Goldman V., Green D.W. (2010). Advances in growth plate modulation for lower extremity malalignment (knock knees and bow legs). Curr. Opin. Pediatr..

[6] Kumar A., Gaba S., Sud A., Mandlecha P., Goel L., Nayak M. (2016). Comparative study between staples and eight plate in the management of coronal plane deformities of the knee in skeletally immature children. J. Child. Orthop..

[7] Masquijo J.J., Artigas C., de Pablos J. (2021). Growth modulation with tension-band plates for the correction of paediatric lower limb angular deformity: Current concepts and indications for a rational use. EFORT Open Rev..

[8] De Brauwer V., Moens P. (2008). Temporary hemiepiphysiodesis for idiopathic genua valga in adolescents: Percutaneous transphyseal screws (PETS) versus stapling. J. Pediatr. Orthop..

[9] Aslani H., Panjavy B., Bashy R.H., Tabrizi A., Nazari B. (2014). The efficacy and complications of 2-hole 3.5 mm reconstruction plates and 4 mm noncanulated cancellous screws for temporary hemiepiphysiodesis around the knee. J. Pediatr. Orthop..

[10] Ballal M.S., Bruce C.E., Nayagam S. (2010). Correcting genu varum and genu valgum in children by guided growth: Temporary hemiepiphysiodesis using tension band plates. J. Bone Jt. Surg. Br..

[11] Raab P., Wild A., Seller K., Krauspe R. (2001). Correction of length discrepancies and angular deformities of the leg by Blount’s epiphyseal stapling. Eur. J. Pediatr..

[12] Radtke K., Goede F., Schweidtmann K., Schwamberger T., Calliess T., Fregien B., Stukenborg-Colsman C., Ettinger M. (2020). Temporary hemiepiphysiodesis for correcting idiopathic and pathologic deformities of the knee: A retrospective analysis of 355 cases. Knee.

[13] Ramseier L.E., Sukthankar A., Exner G.U. (2009). Minimal invasive epiphysiodesis using a modified “Canale”-technique for correction of angular deformities and limb leg length discrepancies. J. Child. Orthop..

[14] Ghanem I., Karam J.A., Widmann R.F. (2011). Surgical epiphysiodesis indications and techniques: Update. Curr. Opin. Pediatr..

[15] Willegger M., Bouchard M., Windhager R., Kolb A., Chiari C. (2022). Epiphysiodesis and hemiepiphysiodesis: Physeal arrest and guided growth for the lower extremity. Orthopade.

[16] Weinmayer H., Breen A.B., Steen H., Horn J. (2022). Angular deformities after percutaneous epiphysiodesis for leg length discrepancy. J. Child. Orthop..

[17] Wiemann J.M., Connor T., Szalay E.A. (2009). Physeal stapling versus 8-plate hemiepiphysiodesis for guided correction of angular deformity about the knee. J. Pediatr. Orthop..

[18] Kulkarni R.M., Ilyas Rushnaiwala F.M., Kulkarni G.S., Negandhi R., Kulkarni M.G., Kulkarni S.G. (2015). Correction of coronal plane deformities around the knee using a tension band plate in children younger than 10 years. Indian J. Orthop..

[19] Morasiewicz P., Leyko P., Tomczyk Ł., Kazubski K. (2024). Do Patient Sex and Age Affect Hemiepiphysiodesis Outcomes?. J. Clin. Med..

[20] Leyko P., Zaborska M., Walczak A., Tomczyk Ł., Pelc M., Mnich A., Operacz R., Morasiewicz P. (2025). Gait Analysis in Patients After Hemiepiphysiodesis Due to Valgus or Varus Knee Deformity. J. Clin. Med..

[21] Kurup J.K.N., Shah H.H. (2020). Hemiepiphysiodesis using 2-holed reconstruction plate for correction of angular deformity of the knee in children. J. Orthop..

[22] Schagemann J., Kudernatsch N., Russlies M., Mittelstädt H., Götze M., Horter M., Paech A., Behnke B. (2022). Prediction of loss of correction after hemiepiphysiodesis for the alignment of lower limb angular deformities. Medicine.

[23] Morasiewicz P., Urbański W., Kulej M., Dragan S.Ł., Dragan S.F., Pawik Ł. (2018). Balance and lower limb loads distribution after Ilizarov corticotomy. Injury.

[24] Ataç A., Akil Ağdere S., Dilek B. (2023). Comparison of the Foot-Ankle Characteristics and Physical and Functional Performance of Racquet Sport Players. Arch. Health Sci. Res..

[25] Arslan M., Görgü S.Ö. (2023). Effect of short-term spinal orthosis and insoles application on cobb angle, plantar pressure and balance in individuals with adolescent idiopathic scoliosis. Clin. Biomech..

[26] Brusa J., Maggio M.C., Giustino V., Thomas E., Zangla D., Iovane A., Palma A., Corsello G., Messina G., Bellafiore M. (2020). Upper and lower limb strength and body posture in children with congenital hypothyroidism: An observational case-control study. Int. J. Environ. Res. Public Health.

[27] Feka K., Brusa J., Cannata R., Giustino V., Bianco A., Gjaka M., Iovane A., Palma A., Messina G. (2020). Is bodyweight affecting plantar pressure distribution in children? An observational study. Medicine.

[28] Morasiewicz P., Dragan S., Dragan S.Ł., Wrzosek Z., Pawik Ł. (2016). Pedobarographic analysis of body weight distribution on the lower limbs and balance after Ilizarov corticotomies. Clin. Biomech..

[29] Suciu O., Onofrei R.R., Totorean A.D., Suciu S.C., Amaricai E.C. (2016). Gait analysis and functional outcomes after twelve-week rehabilitation in patients with surgically treated ankle fractures. Gait Posture.

